# Epidemiology of Sudden Cardiac Death in Rural South India - Insights From The Andhra Pradesh Rural Health Initiative

**Published:** 2011-07-03

**Authors:** Srinivas R Madhavan, Sathish Reddy, Pradeep K Panuganti, Rohina Joshi, Jaya Mallidi, Krishnam Raju, K.Rama Raju, Srinivas Iyengar, K.Srinath Reddy, Anushka Patel, Bruce Neal, Narasimhan Calambur, Harikrishna Tandri

**Affiliations:** 1 CARE Foundation. Hyderabad, India; 2 Byrraju Foundation. Hyderabad, Andhra Pradesh; 3 Centre for Chronic Disease Control. New Delhi, India; 4 The George Institute for International Health. Sydney, Australia; 5 The Johns Hopkins University School of Medicine, Baltimore , USA

**Keywords:** Epidemiology, Sudden Cardiac Death, Rural South India, Andhra Pradesh

## Abstract

**Background:**

Sudden cardiac death (SCD) is a common initial presentation of coronary artery disease (CAD). Despite the growing epidemic of CAD in India, the epidemiology of SCD is largely unknown.

**Objective:**

The objective of the study was to define the prevalence and determinants of sudden cardiac deaths in rural South India.

**Methods:**

Prospective mortality surveillance was conducted in 45 villages (180,162 subjects) in rural South India between January 2006 and October 2007. Trained multipurpose health workers sought to do verbal autopsies within 4 weeks of any death. Detailed questionnaires including comorbidities and circumstances surrounding death were recorded. SCD was adjudicated using the modified Hinkle-Thaler classification.

**Results:**

A total of 1916 deaths occurred in the study population over the 22 month time period and verbal autopsy was obtained in 1827 (95%) subjects. Overall mean age of the deceased was 62 ± 20 years and 1007 (55%) were men. Cardiovascular and cerebrovascular diseases together accounted for 559 deaths (31%), followed by infectious disease (163 deaths, 9%), cancer (126 deaths, 7%) and suicide (93 deaths, 5%).

Of the 1827 deaths, after excluding accidental deaths (89 deaths), 309 deaths (17%) met criteria for SCD. Cardiovascular disease was the underlying causes in the majority of the SCD events (231/309 (75%)). On multivariate analyses, previous MI/CAD (p < 0.001, OR 14.25), hypertension (p < 0.001, OR 1.84), and age groups between 40-60 yrs (p=0.029) were significantly associated with SCD.

**Conclusion:**

Sudden cardiac death accounted for up to half of the cardiovascular deaths in rural Southern India. Traditional cardiovascular risk factors were strongly associated with SCD.

## Introduction

India is the second most populous country in the world with an estimated population of over 1 billion. Rapid industrialization and urbanization have resulted in tremendous growth in the economy over the last decade. Concurrently, India has also seen an exponential rise in prevalence of diabetes, obesity and hypertension- partly related to better life expectancy, lifestyle changes and smoking habits [1,2]. The last decade has also seen significant change in mortality trends with chronic disease replacing infectious disease as the leading cause of death [3] and the World Health Organization has declared India the coronary artery disease (CAD) capital of the world [1]. Sudden cardiac death (SCD), defined, as sudden unexpected death occurring within 1 hour of onset of symptoms, is a common initial manifestation of CAD [4]. The majority of SCD events are attributable to cardiovascular causes even in the absence of a history of cardiac disease [5]. Recently, large epidemiologic studies have reported on the impact of CAD on mortality in India using population-based cohorts, and in selected high-risk groups [6,7]. However, the proportion of cardiovascular deaths that are SCD events and the risk factor profile of the individuals at risk for SCD in India remain largely unknown. The majority of the population lives in rural areas with little or minimal access to health care, with regional differences in disease prevalence and risk factors which are different from the aggregate national data [8,9]. Published data regarding mortality statistics for SCD are of limited applicability as most of the studies were performed in urban settings. The Andhra Pradesh Rural Health Initiative (APRHI) mortality surveillance system that exists in 45 villages in East Godavari District provided a unique opportunity to study SCD incidence and predictors in a rural setting.  The purpose of our study was to define the epidemiology of SCD in rural Andhra Pradesh, India.

## Methods

The data for this study was collected as part of the APRHI, a collaborative effort established in 2002 between the Byrraju Foundation (Hyderabad, India) [10], the Centre for Chronic Disease Control (New Delhi, India) [11], the CARE Foundation (Hyderabad, India) [12], The George Institute for International Health, University of Sydney (Sydney, Australia) [13], and the School of Population Health, University of Queensland (Brisbane, Australia) [14]. Specifically, the verbal autopsy data for this study were collected between January 2006 and 30th September 2007 from 45 villages (population 180,162) in East and West Godavari. The methodologies for data collection used in this study and the validity of the methods have been previously published [3]. The study was approved by the Ethics Committees of the CARE Foundation, Hyderabad, India and the University of Sydney, Australia. Informed consent was obtained from the next of the kin (respondent) prior to the collection of any data.

### Mortality Surveillance System

The construct and the design of the mortality surveillance have been previously published [3]. Briefly, the system was designed to record, and assign causes to all deaths in a defined population. Surveillance was conducted throughout the year on a prospective basis. A multipurpose health worker under the supervision of a field coordinator collected all mortality data from each village using a verbal autopsy tool. The multipurpose health workers were usually natives of the same village, had at least 10 yrs of schooling and were provided with training in the use of the verbal autopsy tool. In addition to the initial training, the MPHW also had re-training sessions at 6 monthly intervals. To ensure complete recording of deaths an information network comprising of the village headman, the Government auxiliary nurse, priests, cremation staff, other community leaders and the village midwife was established in each village. The MPHWs interacted with the information network in addition to their direct interaction with the villagers on a day-to-day basis.

### Verbal Autopsy Tool

The verbal autopsy method was used to determine causes of death.[7] The verbal autopsy method used in this study has been validated [3,15-17,31-33] and has been shown to be useful in developing countries.  The tool consisted of both structured and unstructured questions including a detailed narrative provided by the respondent. The respondent was carefully chosen by the MPHW to be the most reliable source of information based on the proximity of the family member to the deceased relative. To avoid recall bias the interview of the close relative or caregiver of the deceased was sought within 4 weeks of death in the majority of the deaths. Detailed questionnaires including comorbidities and an unstructured narrative on the circumstances surrounding the death were recorded. Medical history of the deceased was collected by the MPHW during the interview process with prompts to inquire about symptoms, treatment and medical procedures undergone by the deceased.  Wherever possible, the data were verified by abstracting information from medical documents held in the home.

### Cause of Death

The cause of death was ascertained based on the data collected by the MPHW. A -trained physician assessed each autopsy questionnaire and selected a cause from a restricted list of diseases derived from the International Classification of Diseases- 10 (ICD-10) [18]. Special algorithms developed for the Registrar General of India's sample registration system were used by the physician in defining the cause of death from the data collected by the MPHW.

### Adjudication of SCD

SCD was adjudicated based on Modified Hinkle-Thaler classification [4,19]. Death was considered to be secondary to SCD if it fell in any one of the four categories: 1) death occurring suddenly and unexpectedly within 1 h of cardiac symptoms in the absence of prior cardiac illness, 2) Death occurring within 1 h of the onset of cardiac symptoms in the setting of stable cardiac disease; 3) Unexpected death during sleep; and 4) death occurring unexpectedly within 24 h after last being seen alive. Two physicians who were trained in Internal Medicine and held Masters Degrees in Public Health made adjudications of SCD from the verbal autopsies. A third physician who is a trained electrophysiologist (HT) supervised the entire adjudication process and resolved disparities in the event of disagreement in the cause of death. All the deaths were adjudicated as SCD or Non-SCD events using the above definition, in addition to sub-classifying the deaths in to the individual ICD-10 diseases. Accidental deaths, suicides and traumatic deaths were not included under sudden deaths.

### Statistical Analyses

All data analyses for research aims were performed using Statistical Analysis Software version 9.2 (SAS 9.2). Cause of death was ascertained based on the verbal autopsy. A p value of < 0.05 was considered to be significant. The cardiac risk factors including gender, age group, diabetes, hypertension, prior MI, alcoholism and smoking were analyzed to determine their association in predicting SCD. The significant variables were then included in a multivariate analysis.

## Results

A total of 1916 deaths occurred in the population over the 22 month time period of the study. The population had a literacy of approximately 54% and a mean household monthly income of US$ 50. The majority of the population worked as laborers in the agriculture and aquaculture industry. Of the 1916 deaths, verbal autopsy was recorded in 1827 (95%) subjects. Table 1 shows the baseline characteristics of the study population. Overall mean age of the deceased was 62 ± 20 years and 1007 (55%) were men. The majority of deaths (79%) occurred at home and women were more likely to die at home compared with men (p<0.01).

The majority of both sudden and non-sudden deaths occurred at home. More importantly 85% of the sudden and non-sudden deaths were witnessed with the responder present at the time of the death with the spouse or child of the deceased present at the time of death in most cases.

In the entire cohort, cardiovascular and cerebrovascular diseases together accounted for 559 deaths (31%), followed by infectious disease (163 deaths, 9%), cancer (126 deaths, 7%) and suicide (93 deaths, 5%). Of the 1827 deaths, after excluding accidental deaths (89 deaths), 309 deaths (17%) met criteria for SCD.     Of the 1827 deaths, 309 deaths (17%) met criteria for SCD and were adjudicated as SCD events.

### Predictors of SCD

**Figure 1:**
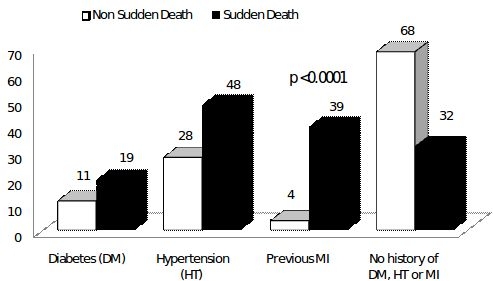
Prevalance of Cardiovascular risk factors among sudden and non sudden deaths.

Shown in Figure 1 is the prevalence of cardiovascular risk factors among sudden and non-sudden deaths. Subjects experiencing SCD were significantly more likely to have hypertension, diabetes and a history of myocardial infarction/coronary artery disease (p<0.01 for all).  A third of SCD events (96/309) were preceded by chest pain. Cardiovascular disease was the underlying cause in the majority the deaths that were adjudicated as SCD events (231/309 (75%)), followed by cerebrovascular disease and other unspecified causes accounting for the remainder of deaths (Figure 2a). In contrast, cardiovascular disease accounted for only 11.4% (173/1518) of the 1518 non SCD deaths. Cerebrovascular disease, cancer, suicide and infectious causes accounted for (10%, 8%, 6% and 11% respectively) of the non-SCD deaths (Figure 2b).

**Figure 3:**
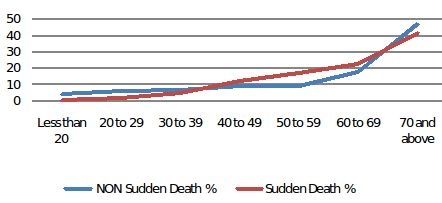
Age Distribution non sudden and sudden deaths

Table 2 presents data on the measures of association of the different covariates in predicting SCD.  On univariate analyses, gender, age between 40-70 yrs, diabetes, hypertension, and prior MI/CAD were significantly associated with SCD. The association of age with SCD is shown in Figure 3. The proportion of deaths attributable to SCD and non-SCD were similar until the age of 40 when there was a significant increase in SCD events, which declined after the age of 70. On multivariate analyses that included the significant univariate predictors, previous MI/CAD, hypertension and age groups between 40-60 yrs were significantly associated with SCD.

## Discussion

Our study has several important findings. Firstly, sudden cardiac death appears to account for over half of all cardiovascular deaths in a large population based cohort in rural South India. Secondly, the risk of SCD appears to be highest between the ages of 40-60 yrs. Traditional cardiovascular risk factors such as prior MI, and hypertension are independently associated with SCD. Finally, literacy, gender, diabetes, and smoking habit do not seem to affect SCD.

India is in the middle of a major economic and industrial transition. The associated lifestyle and dietary changes have led to rise in smoking habits, hypertension, obesity, diabetes and in turn, coronary artery disease (CAD) [1,2]. CAD accounts for 1.5 million deaths annually and several studies have shown younger age of onset of CAD in India [29,30]. A significant proportion of deaths in CAD are possibly sudden cardiac deaths (SCD), although the exact prevalence of SCD is unknown. The reported prevalence of SCD in Western populations has ranged between 10-12% [20-22], whereas the prevalence in our study was 17%.  The difference in the prevalence can be in part due to the high prevalence of cardiovascular risk factors in India and age at manifestation of the risk factors between the two groups [23]. Reports from South India have documented a high prevalence of hypertension (27.2%), diabetes mellitus (16.3%) and hyperlipidemia (30.3%). Further the prevalence of multiple cardiovascular risk factors (smoking, hypertension, dyslipidemias, diabetes and metabolic syndrome) rapidly escalates by age of 30-39 years. Moreover, lack of primary prevention and risk factor modification practices might amplify the differences seen in the prevalence between the Western and South Indian cohorts.

The higher prevalence of SCD might also be due to absence of the "chain of survival" i.e. emergency medical services in rural areas, and lack of awareness of the symptoms of myocardial infarction. Of note, the respondents reported chest pain shortly before death, occurring in a third (31%) of the individuals who had SCD events. The risk of SCD is high in the setting of an acute myocardial infarction and revascularization, by means of thrombolysis and/or primary angioplasty might affect the risk of SCD. The lack of access to medical care is also reflected by the fact that the majority of SCD subjects died at home, despite the fact that a substantial proportion of patients had no antecedent illness and SCD were unexpected. Thus the differences in health care infrastructure, access and utilization may have a role in influencing the prevalence of SCD in rural India.

The prevalence of cardiovascular risk factors such as hypertension, diabetes, and CAD was significantly higher in the SCD group compared with the non-SCD group. These data are consistent with prior reports that have shown strong association between coronary artery disease and SCD. In fact the epidemiology of SCD to a great extent parallels that of CAD. Prior autopsy studies have revealed coronary occlusion in up to 80% of SCD events [5]. It is thought that acute coronary occlusion due to plaque rupture creates an ischemic substrate with a high risk for ventricular fibrillation. Aggressive risk factor modification and life style changes for primary prevention of CAD have been shown to reduce the incidence of sudden cardiac death. Two thirds of the patients experiencing SCD event had at least one cardiovascular risk factor identified in our study suggesting a possible role for preventive strategies targeting hypertension and diabetes in reducing the SCD prevalence.

Smoking has been independently associated with SCD [25]. Smoking cessation has been shown to reduce the likelihood of sudden death. It is also known that once CAD occurs the independent effect of smoking on SCD decreases [26-28]. We, however, did not find an association between smoking and SCD in our study. Smoking habit may have been underreported due to social taboos, however, the exact reasons for this are unclear and these results need to be validated in subsequent studies.

### Clinical Implications

Our study highlights the high prevalence of SCD in rural India. Given the higher prevalence of coronary risk factors in urban settings, it is conceivable that the prevalence is even higher in the urban population. The majority of SCD events were associated with treatable coronary risks such as hypertension and diabetes. Aggressive risk factor modification in this population may favorably affect SCD related mortality although this hypothesis needs to be tested. Improving access to emergency care, educating the public on symptoms of myocardial infarction and basic cardiopulmonary resuscitation (CPR) practices have proven to be effective in the Western world and SCD in India is unlikely to be an exception. Although not specifically sought during verbal autopsy data collection, the lack of mention of any form of resuscitation in all the deaths analyzed was striking. The majority of SCD events occurred at home and were witnessed in 85% of cases This is not surprising, since CPR practices are virtually non-existent outside hospital settings and awareness of SCD is poor among the general public, more so in rural regions. The results of our study underscore the need for measures to reduce the burden of SCD and improving outcomes following an SCD event by way of establishing the "chain of survival", including public education on CPR practices.

### Limitations

The verbal autopsy tool for our study was not specifically designed to investigate SCD events. Further, the data regarding risk factors were collected from the respondents, which lends itself to error in reporting. Even though the MSWs conducted interviews of the respondents at the earliest possible time, with in 4 weeks of death, there is a potential chance of recall bias. However, random error in collection of data about risks is likely to result in systematic underestimates of the associations with outcomes and not over estimates. Nevertheless, the adjudication of SCD was based solely on the chronology of events leading to the death, which was described in great detail in the unstructured narrative by the respondent. The exact underlying cause of sudden death could not be differentiated in some cases, which is a limitation of the study. The MPHWs received extensive training to ensure accurate collection of data and unbiased recording of the chronology of events surrounding death as narrated by the respondent. MPHW were specifically trained to extract risk factor information via structured questions. In addition, the MPHWs used a symptom list and enquired about treatment and medical procedures performed and also verified available and associated documents. More importantly, the accuracy of data collection and the adjudication that followed have been previously verified [3]. Another important limitation is that data regarding physical activity, obesity and hyperlipidemia, which may have a bearing on cardiovascular disease, were not collected in this study.

In conclusion, SCD appears to be a significant mode of death in rural India and accounts for more than half of the cardiovascular deaths. The SCD prevalence is higher than in Western countries, probably related to higher prevalence of cardiovascular risk factors, lack of preventive care and inadequate health care access. More importantly, there is a strong association between traditional CAD risk factors and SCD events. Primary prevention strategies aimed at control of hypertension and diabetes might impact the prevalence of SCD.

## Figures and Tables

**Figure 2a:**
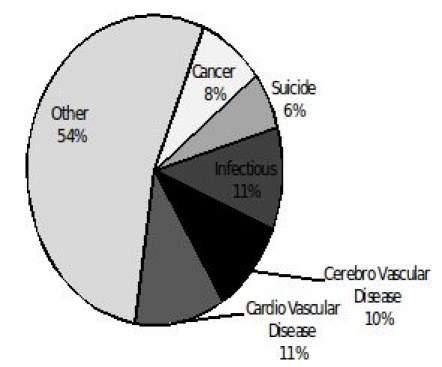
Chief underlying cause of death in non-sudden death

**Figure 2b:**
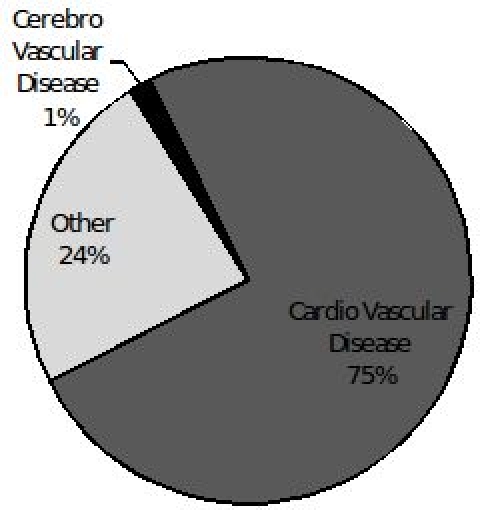
Chief underlying cause of death in sudden death

**Table 1:**
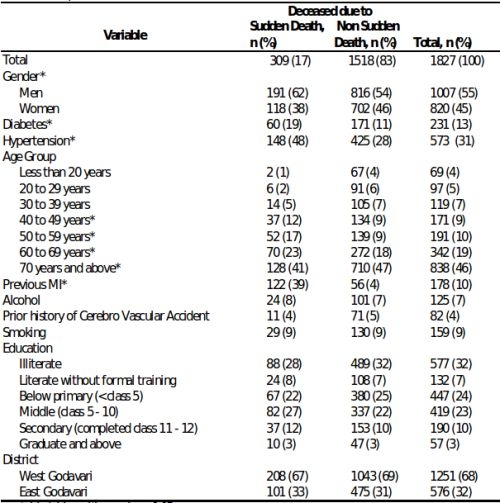
Comparison of characteristics of deceased due to sudden and non sudden death

* Variables with p value < 0.05 % Percentage approximated to the nearest whole number

**Table 2:**
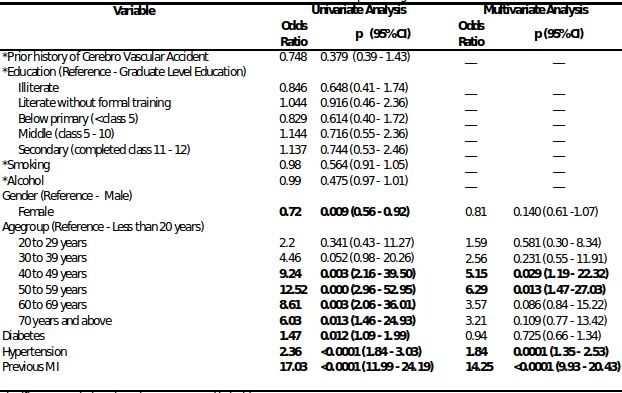
Covariates and their measures of association in predicting Sudden Cardiac Death (SCD)

Significant associations have been presented in bold * Variables not included in multivariate analysis
